# Protocol for PMA-Ethiopia: A new data source for cross-sectional and longitudinal data of reproductive, maternal, and newborn health

**DOI:** 10.12688/gatesopenres.13161.1

**Published:** 2020-09-09

**Authors:** Linnea Zimmerman, Selam Desta, Mahari Yihdego, Ann Rogers, Ayanaw Amogne, Celia Karp, Shannon N. Wood, Andreea Creanga, Saifuddin Ahmed, Assefa Seme, Solomon Shiferaw

**Affiliations:** 1 Department of Population, Family, and Reproductive Health, Johns Hopkins Bloomberg School of Public Health, Baltimore, MD, 2105, USA; 2 PMA-Ethiopia, Addis Ababa University, Addis Ababa, Ethiopia; 3 Department of International Health, Johns Hopkins Bloomberg School of Public Health, Baltimore, MD, 21205, USA; 4 Department of Gynecology and Obstetrics, Johns Hopkins Medicine, Baltimore, MD, 21205, USA; 5 School of Public Health, Addis Ababa University, Addis Ababa, Ethiopia

**Keywords:** Ethiopia, family planning, maternal health, cohort, survey

## Abstract

**Background: **Performance Monitoring for Action Ethiopia (PMA-Ethiopia) is a survey project that builds on the PMA2020 and PMA Maternal and Newborn Health projects to generate timely and actionable data on a range of reproductive, maternal, and newborn health (RMNH) indicators using a combination of cross-sectional and longitudinal data collection.

**Objectives: **This manuscript 1) describes the protocol for PMA- Ethiopia, and 2) describes the measures included in PMA Ethiopia and research areas that may be of interest to RMNH stakeholders.

**Methods: **Annual data on family planning are gathered from a nationally representative, cross-sectional survey of women age 15-49. Data on maternal and newborn health are gathered from a cohort of women who were pregnant or recently postpartum at the time of enrollment. Women are followed at 6-weeks, 6-months, and 1-year to understand health seeking behavior, utilization, and quality. Data from service delivery points (SDPs) are gathered annually to assess service quality and availability.  Households and SDPs can be linked at the enumeration area level to improve estimates of effective coverage.

**Discussion: **Data from PMA-Ethiopia will be available at www.pmadata.org.  PMA-Ethiopia is a unique data source that includes multiple, simultaneously fielded data collection activities.  Data are available partner dynamics, experience with contraceptive use, unintended pregnancy, empowerment, and detailed information on components of services that are not available from other large-scale surveys. Additionally, we highlight the unique contribution of PMA Ethiopia data in assessing the impact of coronavirus disease 2019 (COVID-19) on RMNH.

## Background

Ethiopia has one of the highest neonatal mortality rates (NMR; 28 per 1,000 live births) and maternal mortality ratios (MMR; 401 deaths per 100,000 live births) in the world according to the most recent United Nations estimates ^1, 2^. Despite recent progress in reducing both maternal and neonatal mortality ^3^, significant challenges remain to ensure coverage of high impact interventions for maternal and newborn health; for example, as of 2017, only 28% of deliveries were conducted by a skilled birth attendant and only 13% of infants received postnatal care within two days of delivery ^4^.

Compounding the issue of low coverage of services is the challenge of quickly, effectively, and accurately monitoring the coverage of interventions. The WHO Expert Review Group’s 2012 report found that of the 75 countries that collectively account for 95% of all deaths among women and children, which includes Ethiopia, only 11 had recent data on all eight coverage indicators recommended for global monitoring (i.e. met need for contraception, antenatal care coverage, antiretroviral prophylaxis among HIV-positive pregnant women, skilled birth attendance, postnatal care, exclusive breastfeeding for six months, three doses of DPT vaccine, and antibiotic treatment for childhood pneumonia) ^5^. Though the percent of countries with coverage data has increased in recent years, evidence suggests that measures of service contact alone (e.g. antenatal care, postnatal care) without greater detail of the quality and comprehensiveness of services, underestimate true gaps in service coverage ^6^.

In 2014 and 2016, two large scale collaborative projects between Addis Ababa University (AAU) and Johns Hopkins Bloomberg School of Public Health (JHBSPH), funded by the Bill and Melinda Gates Foundation (BMGF), were launched in Ethiopia to generate timely data on reproductive, maternal, and newborn health (RMNH). The first project, Performance Monitoring and Accountability 2020 (PMA2020), provided annual nationally representative estimates of key indicators related to family planning between 2014 and 2018 ^7^. The second project, PMA Maternal and Newborn Health (PMA-MNH), was conducted in Southern Nations Nationalities and People’s (SNNP) region to provide information about coverage of specific high-impact interventions and the quality of maternal and newborn health (MNH) services. PMA-MNH was a longitudinal study that enrolled pregnant women in SNNP and followed them through six months postpartum to generate data with minimal recall bias and improved measurement of the continuum of reproductive, maternal, newborn, and child health care. Both projects identified gaps in RMNH coverage and service utilization and highlighted significant disparities among populations, including regional disparities in family planning use and quality and disparities in the quality and coverage of antenatal and postnatal care services by residence, parity, and wealth ^8, 9^.

The generation of coverage estimates from both surveys were met with great interest from the Federal Ministry of Health (FMoH), while the identification of significant disparities across and within regions highlighted the need to explore the determinants of MNH care services in greater detail. The FMoH and BMGF thus supported the realignment of the PMA2020 and PMA-MNH grants to create PMA-Ethiopia. PMA-Ethiopia was developed to provide national estimates of key RMNH indicators and generate new evidence on the barriers and facilitators to use of health services through its longitudinal study design.

In addition to broadening the scope of the project to include both family planning and RMNH care, PMA-Ethiopia is dedicated to improving data utilization, particularly within the FMoH. A Project Advisory Board (PAB) was formed in January of 2019, chaired by the State Minister of Health, and composed of representatives from the FMoH, professional associations (e.g. Ethiopian Society of Obstetric and Gynecologists), multilateral organizations (e.g. UNICEF, UNFPA), non-governmental organizations (e.g. Marie Stopes International, Pathfinder, Engender Health), and donors (e.g. BMGF, DfID) that actively provide and/or support RMNH programs in Ethiopia. The PAB provided critical input during the survey design and development stage to inform the content and scope of the survey and will review all preliminary results prior to dissemination to offer critical clinical and programmatic perspectives about the data. The PAB further advises PMA-Ethiopia about indicators and analyses that require more in-depth exploration, encourages data utilization within their organizations, and provides feedback on dissemination strategies to effectively and rapidly share data from PMA-Ethiopia with relevant stakeholders. With a continued focus on generating high-quality and rapid data, PMA-Ethiopia is uniquely positioned as a critical data source within the country’s data architecture.

This manuscript describes the protocol for PMA-Ethiopia, including methodology and key development activities and describing the measures included in PMA Ethiopia and research areas that may be of interest to RMNH stakeholders.

### Objectives

PMA-Ethiopia builds on the previous success of both the PMA2020 cross-sectional surveys and the PMA-MNH panel survey. With the overall goal of identifying gaps in RMNH care services, the specific objectives of the PMA-Ethiopia project are to:

1. Generate nationally and regionally representative data on family planning indicators and factors associated with use, at both the household and service delivery point level;
2. Evaluate coverage and comprehensiveness of the continuum of RMNH services in the first year postpartum in six regions, at both individual woman and service delivery point levels;
3. Identify factors at the service delivery point, community, partner, and individual levels that are associated with RMNH service utilization.

A fourth objective was added in May 2020 in response to the coronavirus disease 2019 (COVID-19) pandemic.

4. To assess whether key maternal and newborn health outcomes have been adversely affected by the COVID-19 pandemic, including health-seeking behaviors related to antenatal, delivery, and postnatal care, such as postpartum family planning, and immunizations for infants.

## Study design

**Figure 1.**
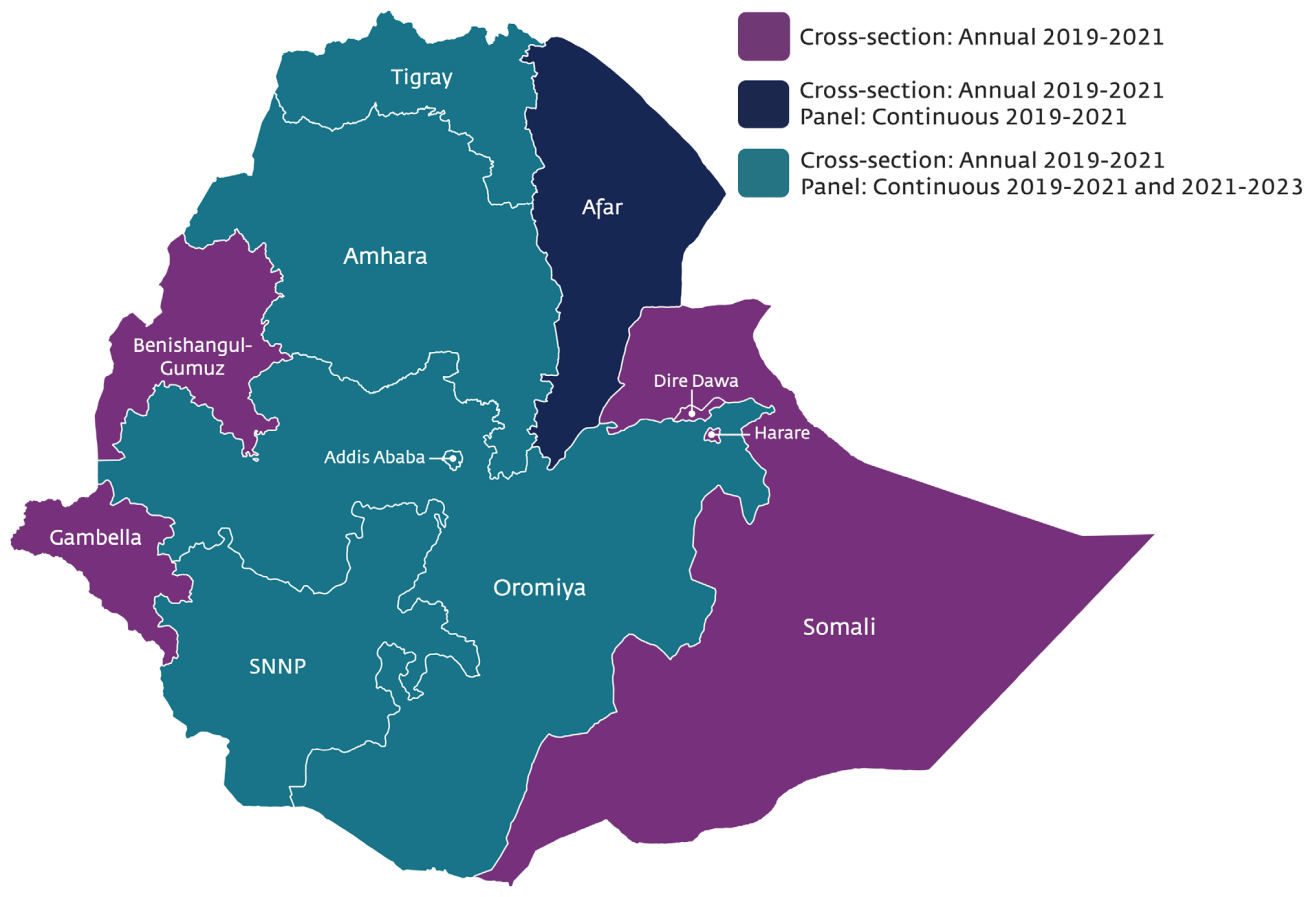
Map demonstrating survey coverage and activities of Performance Monitoring for Action (PMA) Ethiopia.

PMA-Ethiopia is composed of three distinct study activities, including 1) annual cross-sectional surveys of women aged 15–49 years; 2) longitudinal surveys of women who are currently pregnant or recently postpartum; and 3) annual service delivery point (SDP) surveys of health facilities ( Figure 1).

The **cross-sectional survey** of all women ages 15–49 years is a nationally representative household survey, focusing on family planning and reproductive health behaviors and outcomes and will be repeated annually from 2019 to 2022. A **panel of** all women who are pregnant or less than 6 weeks postpartum at the time of enrollment in the survey will be followed up at 6-weeks, 6-months, and 1-year postpartum through separate surveys. The panel is conducted in six regions that collectively represent 90% of the population in Ethiopia; Addis Ababa, Afar, Amhara, Oromia, SNNP, and Tigray. The panel methodology will be repeated for two cohorts of women: the first enrolled in the fall of 2019 and the second will enroll in the fall of 2021. Supplemental funds allowed for the inclusion of Afar region in the first cohort of the panel survey only. Finally, the **service delivery point (SDP) survey** includes public and private facilities that serve the identified enumeration areas (EAs) for the household survey. The survey focuses on facility readiness for offering essential RMNH services, while also capturing additional quality of care provision aspects. The SDP survey will be repeated annually from 2019 to 2022.

While some aspects of each survey differ (e.g. listing, eligibility, sample design), they share many common aspects of design and fieldwork (e.g. Open Data Kit (ODK) programming, training). Procedures that varied between surveys are described by survey when relevant.

### Sample design

***Cross-section.*** In order to assess trends since the original PMA2020 survey, the PMA-Ethiopia survey used the modern contraceptive prevalence rate (mCPR) among all women as the key indicator to derive sample size. We calculated the required number of households and women necessary to generate a 5% margin-of-error at the regional level based on the sample size calculation formula of Wilson’s method for asymmetric proportions ^10^. Previous estimates of mCPR from the PMA2020 Ethiopia 2018 survey were used for five regions (Tigray, Amhara, Oromia, SNNP and Addis Ababa), while estimates of mCPR from the Ethiopian Demographic and Health Survey (EDHS 2016) were used for the remaining regions ^4, 11^.

Detecting and tracking change over time in regional-level estimates is a top priority for the FMoH, thus, PMA-Ethiopia made the decision to not reduce the sample size within any region when redesigning the survey from PMA2020 to PMA-Ethiopia. Sample sizes within each region were calculated as described above; if the original sample size from the PMA2020 survey was larger than the new estimate, the original sample size was retained and the urban/rural distribution was updated to reflect the 2019 urban/rural population distribution estimated by the Ethiopian Central Statistical Agency (CSA). If the updated sample size was larger than the original PMA2020 survey, the updated estimate was used.

All EAs were drawn with probability proportional to size within the strata as noted in Table 1. For the regions of Tigray, Amhara, Oromia, and SNNP, EAs were drawn separately from urban and rural strata within the region. For the regions of Afar, Somali, Benishangul-Gumuz (BG), Gambella, and Harari, the EAs were randomly selected with probability-proportional-to-size from within the region, without urban/rural stratification. Addis is exclusively urban and thus all EAs were drawn from within the region without stratification. In total, 265 EAs were selected. Table 1 identifies the total number of EAs per region and the anticipated number of women to be interviewed in each cross-sectional survey.

**Table 1.** Tb1 SampleDesc.

Region	Sampling strata	Total EAs	Anticipated number of women
Tigray *	Region + urban/rural	34	1052
Afar *	Region	13	402
Amhara *	Region + urban/rural	49	1516
Oromia *	Region + urban/rural	52	1610
Somali	Region	6	186
BG	Region	9	278
SNNP *	Region + urban/rural	47	1455
Gambella	Region	11	341
Harari	Region	11	341
Addis *	Region-Urban	22	681
Dire Dawa	Region	11	341
**Total**		**265**	**8203**

Notes: *Regions that also complete the panel survey;

**Figure 2.**
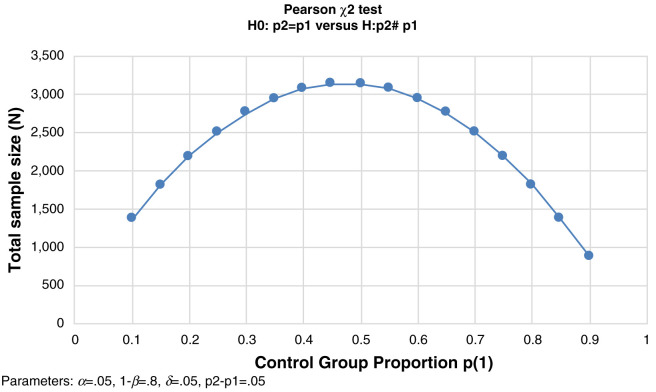
Estimated total sample size for a two-sample proportions test to detect 5% difference.

***Panel.*** EAs selected for the cross-section also serve as the EAs for the panel. The panel is designed to detect differences in health behaviors between groups “exposed” to various components of the Ethiopian health system (e.g. to detect differences in receipt of postnatal care by two days postpartum between women who were contacted by an HEW during antenatal care and those who were not). The largest sample size needed to detect a difference of 5% between groups of interest (alpha of 0.05 and power of 0.80) is 3,130, as shown in Figure 2 below. As the prevalence of the majority of RMNH indicators in Ethiopia are significantly less than 50%, sample sizes of approximately 2,500 and 3,000 are sufficient to detect differences of 5%.

Based on data from previous PMA2020 rounds, we assumed that approximately 10% of women age 15–49 years were pregnant or had given birth within 6 weeks. Thus, we needed to screen between 25,000 and 30,000 women. Assuming approximately 0.98 women ages 15–49 years per household and an average EA size of 175 (both estimates based on previous PMA rounds), we estimated that we would need approximately 175 EAs in the panel regions to achieve this sample size. Our final sample size of 217 EAs in the panel regions was thus deemed adequate for detecting a 5% difference between groups for the majority of RMNH indicators.

***Service Delivery Point (SDP).*** Once EAs were identified, we obtained a list of all public and private health facilities from the local district health offices that included all health posts, health centers, and primary level hospitals in corresponding districts. While private health facilities are still relatively rare in Ethiopia, information about the RMNH services that private facilities provide is also collected. To sample private health facilities, the list of all private health facilities in each kebele, the lowest level administrative unit in Ethiopia which generally includes about 5 EAs, is reviewed. A maximum of three private SDPs within the kebele boundaries are randomly selected for interview, aligning with the previous PMA2020 protocols ^7^.

All sample size calculations were conducted using Stata version 16 ^12^.

## Questionnaire development

All questionnaires were developed to ensure alignment on priority indicators for the FMoH, PAB, and BMGF strategy teams (see extended data ^13– 15^). The 2016 PMA-MNH survey served as a base for the panel survey, with previous PMA2020, the global Performance Monitoring for Action (PMA) surveys, and Demographic and Health (DHS) surveys used as the foundation for the cross-sectional measures. The development of the SDP questionnaire was collectively informed by prior SDP surveys within PMA2020, DHS Service Provision Assessment (SPA) and WHO Service Availability and Readiness Assessment (SARA) survey tools. For each survey, the JHBSPH and AAU teams discussed inclusion of additional items for research and measurement innovation. For each data collection round, questionnaires integrating the retained questions from previous survey rounds in addition to new items will be drafted and circulated to the FMoH, BMGF, and the PAB for feedback.

Draft cross-section, panel, and SDP questionnaires for the first round of data collection were piloted in EAs and SDPs in Addis Ababa and the surrounding Oromia zones in May 2019. These EAs and SDPs were not sampled for the actual survey. During the pilot exercise, the questionnaires were reviewed in detail by the field team for coherence, wording, and option choices. The field team also conducted a series of practice interviews with both rural and urban respondents and health facilities outside of the study area. Three separate cognitive interview guides were developed to ensure comprehension of new survey items for women across the reproductive life-course (i.e. nulliparous and parous women, users and non-users of family planning, pregnant and postpartum women). The family planning cognitive interview focused on perceptions and experience of contraceptive side effects, knowledge of abortion laws, reproductive coercion, and community norms surrounding contraceptive use. The pregnancy interview covered pregnancy intentions, questions related to pregnancy autonomy and agency, and attitudes towards child vaccinations, while the postpartum interview focused on respectful maternity care during labor and delivery, help-seeking behaviors, and postpartum family planning (PPFP) counseling and use. An additional round of cognitive interviewing occurred in July 2019 specifically to test revised respectful maternity care items ^16^.

## Measures

The list of measures below in Table 2, provides an overview of the key study measures captured within each of the three surveys: cross-section, panel, and SDP. In addition to standard coverage indicators, the surveys included a number of innovative measures to amplify the utility of PMA-Ethiopia data to answer critical questions in the field of RMNH.

**Table 2.** Survey tools and their associated indicators.

Survey Tool	Indicator
**Cross-section** Sample: Women aged 15–49	*Family planning use and dynamics*
	Contraceptive prevalence rate (CPR)
	Modern contraceptive prevalence rate (mCPR)
	Method mix
	Unmet need for family planning
	Contraceptive discontinuation
	Reasons for non-use of contraception
	*Psychosocial factors*
	Existence of choice for family planning (proxy for empowerment)
	Experience/Fear of contraceptive side effects
	Community norms about family planning
	Community norms about pregnancy
	Exposure to messaging about family planning
	Pregnancy intentions *(Source: Demographic and Health Surveys measure; London* *Measure of Unplanned Pregnancy; affective measure)*
	Knowledge of abortion laws
	*Partner dynamics*
	Reproductive coercion * (Revised Conflict and Tactics Scale)*
	Male partner involvement in decision-making about family planning
	*Experience of care*
	Place family planning was received
	Cost of family planning service
	Provider bias in contraceptive counseling
	Quality of contraceptive counseling
**Panel** Sample: Currently pregnant and recently postpartum women	*Antenatal care (ANC)*
	Receipt of any ANC during pregnancy
	Timing of ANC initiation
	Number of ANC visits received
	Components of ANC received
	Counseling on pregnancy-related care at ANC (e.g., place of delivery, delivery by a skilled birth attendant, danger signs)
	Counseling on postpartum family planning (PPFP) at ANC
	Counseling on nutrition and diet
	Participation in 1-to-5 groups
	Use of insecticide-treated nets during pregnancy
	*Labor and delivery (L&D)*
	Experience of complications during pregnancy
	Contact with Health Extension Workers (HEWs) about labor
	Place of delivery
	Skilled birth attendant (SBA) at delivery
	*Psychosocial factors*
	Existence of choice for pregnancy (proxy for empowerment)
	Pregnancy intentions * (Source: Demographic and Health Surveys measure; London Measure of* *Unplanned Pregnancy; affective measure)*
	Intention to use postpartum contraception
	*Partner dynamics*
	Reproductive coercion * (Revised Conflict and Tactics Scale)*
	Intimate partner violence (IPV) during pregnancy
	Male partner involvement in decision-making about pregnancy-related care and family planning
	*Postpartum experiences and behaviors*
	Resumption of sexual activity
	Use of postpartum family planning (PPFP)
	Unmet need for postpartum family planning
	Use of Lactational Amenorrhea Method (LAM)
	Provider bias in postpartum contraceptive counseling
	Discontinuation of contraceptive methods
**Service delivery point** Sample: Survey completed by facility in-charge at all public and three private facilities per survey enumeration area	*Facility readiness*
	Infrastructure (e.g., electricity, water, toilets, telephone, blood bank)
	Staffing by provider type
	Stock availability of contraceptive commodities
	Stock availability of life-saving medicines
	Service availability for specific contraceptive methods
	Service availability for ANC, L&D, postnatal care (PNC), neonatal intensive care (NICU), postabortion care (PAC), family planning, etc.
	Service availability for implant/IUD removal
	Service and stock availability of immunizations
	*Referral systems*
	Existence of referral forms for referred patients
	Availability of emergency transport vehicle
	Existence of designated liaison officer for referrals
	Existence of functional mechanism for recording and sharing outcomes of referred cases
	*Health management information systems (HMIS)*
	Existence and type of functional mechanism for summarizing key outcome data (e.g., manual/paper-based, DHIS2, eCHIS)
	Existence of functional mechanism for reporting data on maternal deaths to the Maternal and Perinatal Death Surveillance Response
	Generation and frequency of reports for zonal, district, regional or national HMIS monthly or more often
	Receipt of action-oriented recommendations from HMIS data
	Existence and use of maternal death review
	*Quality of care*
	Provision of care dimension of WHO’s quality of care framework
	Existence and use of performance monitoring team (PMT)
	Use of participatory performance review meetings
	Existence of national guidelines and protocols for care
	Use of iCCM/IMNCI registration books for sick infants and children
	*Standard practices for:*
	ANC clients
	Infection control precautions
	Emergency obstetric and essential newborn services
	Safe abortion counseling and referral
	Safe abortion and postabortion care visits
	Storage of family planning commodities
	*Facility caseloads*
	Delivery volume
	Cesarean delivery volume
	Safe abortion and postabortion care clients
	Family planning (by method)
	*Facility statistics*
	Maternal deaths
	Stillbirths
	Very early neonatal deaths
	Neonatal deaths

*Italicized text indicates sub-topic area.*

### Cross-section

The cross-sectional survey includes a short household survey, administered in each of the 35 households selected for the survey, and a longer female questionnaire, administered to every eligible female age 15–49. The female questionnaire includes questions about women’s use of contraception, experience, and perceptions of side effects related to contraceptive use, contraceptive autonomy, experience of reproductive coercion, community attitudes surrounding contraception and pregnancy, and interactions with healthcare providers related to family planning services, including whether women felt pressured by providers to use specific methods (provider bias). We also assess women’s knowledge of abortion laws and accessibility in Ethiopia.

### Panel

We leverage the cohort design of PMA-Ethiopia to investigate a number of measures specific to women’s health and well-being across the continuum of care. This includes information gathered only at specific stages of pregnancy or the postpartum period (e.g. delivery, immediate postnatal care, vaccinations) and repeated measures to examine changes over time (e.g. fertility intentions). Information on individual, partner, and community factors were gathered at baseline to assess how different levels of influence affect care-seeking for RMNH.

To improve our understanding of individual and partner influences on health seeking and RMNH outcomes, questionnaires include a range of measures that we hypothesize influence women’s care seeking. These include the Conflicts and Tactics Scale (CTS-2) to measure violence during pregnancy ^17^ and multiple measures designed to assess unintended pregnancy, including the London Measure of Unintended Pregnancy (LMUP) ^18^. Measures from the Women’s and Girls’ Empowerment in Sexual and Reproductive Health (WGE-SRH) index are also included, including the pregnancy existence of choice sub-scale and the sexual existence of choice sub-scale, which measures constraints on the choice to become pregnant and to have sex, respectively ^19, 20^. Similar to the cross-section, the panel study includes measures of reproductive coercion, community attitudes about pregnancy, and provider interactions for post-partum family planning services.

### Service Delivery Point

The SDP survey focuses on measuring facility-based indicators of readiness to provide care across the RMNH continuum. In addition to collecting information on the availability of services and commodities, the SDP survey includes specific questions to assess facility preparedness to remove contraceptive implants, utilize health management information systems (HMIS) and referral networks with other facilities, and use Ethiopia’s integrated community case management of childhood illness (iCCM) and integrated management of newborn and child illness (IMNCI) registers to monitor the health trajectories of sick children. The SDP also collects information about aspects of the quality of health services and use of performance monitoring teams (PMTs) to regularly review facility outputs and outcomes. Finally, as SDPs are included in the sample because they serve the selected EAs, SDPs and household data can be linked via EA identifiers in both the household and SDP datasets to provide a detailed picture of the service delivery environment available to households.

## Field preparation

### ODK Programming

All of the questionnaires are programmed using ODK, an open source software for collecting and managing data. The PMA-Ethiopia team has been using this software since 2014 as part of PMA2020. Prior to data collection, all forms undergo multiple quality checks to ensure that they accurately reflect the paper questionnaire — using the same word-for-word text, question order, and skip patterns — and that the ODK forms are “bug free”.

### Training

Prior to the start of the first round of data collection, PMA-Ethiopia held a series of consecutive trainings of field staff. The first was a two-week training-of-trainers (TOT) of field supervisors and regional coordinators who then served as master trainers at the resident enumerator (RE) trainings. Each RE training session was two weeks and included in-depth review of survey protocols, questionnaire content, and interview skills. In addition to classroom exercises, field staff trainings included three days of field exercises, during which participants practiced using the tools on the phone. The trainings were conducted primarily in Amharic. In addition, some small group sessions were conducted in Afan Oromo and Tigrinya. At the TOT, staff and faculty from JHSPH led the training, with support from AAU project staff, while later RE trainings were led by the AAU team and supported by JHBSPH. Additional trainings will be held throughout the course of the project.

## Survey implementation

### Listing and census

In the six regions in which the cross-section and panel are implemented, a full census of the selected EAs was undertaken, listing the names, sex, and ages of all household members. Women age 15–49 within each household were consented and screened for eligibility into the panel (described further below). Upon completion of the census, it served as the sampling frame for the cross-section and 35 households within each EA were randomly selected. In the remaining regions, a household listing was conducted, identifying all households but not identifying individual household members. From this household list, 35 households were randomly selected for the cross-section.

The census and listing frames will be supplemented with new households annually prior to the selection of households for the cross-section. The census will be completely redone in 2021 prior to the baseline of the second cohort of pregnant/recently postpartum women.

### Eligibility, ethics and consent

***Ethical approval.*** PMA Ethiopia received ethical approval from Addis Ababa University, College of Health Sciences (AAU/CHS) (Ref: AAUMF 01-008) and the Johns Hopkins University Bloomberg School of Public Health (JHSPH) Institutional Review Board (FWA00000287).

***Cross-section.*** A brief household questionnaire is administered in every household selected for the cross-section. Consent for the household questionnaire can be granted by any competent member of the household. The household questionnaire is used to collect data on age, sex, and marital status for all usual members of the household or visitors who slept in the household the night before and includes information necessary to construct a wealth index from common assets. From the household roster, all women age 15–49 years are identified and approached for consent to participate in the individual female questionnaire. The National Research Ethics Review Guidelines in Ethiopia considers women age 15–17 as able to consent for themselves when data collection covers sensitive topics, including sexual and reproductive health, so no parental consent was required ^21^. Once identified as eligible, the RE describes the purpose of the study and reads the approved consent language. All consents (including panel and SDP, described further below) are provided as oral consent per guidance from the National Research Ethics Review Guidelines. Based on this guidance and from the IRB on record, written consent is not required in areas of low literacy or when data collection does not include invasive procedures (e.g. biospecimen collection). Data collection procedures were approved by the AAU and JHBSPH Institutional Review Boards.

***Panel.*** All women age 15–49 years who are identified in the census are consented and screened for eligibility. Women are eligible for the panel if they are regular members of the household, including women staying at their parental home for the delivery and postpartum period, and self-identified as currently pregnant or less than six weeks postpartum. At screening, the RE explains the purpose of the survey and cadence of interviews. If the woman consented, a household questionnaire, followed by a baseline questionnaire are conducted, resulting in baseline data collected across a range of gestational ages, from less than one month pregnant to six weeks postpartum.

For women who are pregnant at baseline, their approximate gestational age is used to estimate date of delivery. REs contact the respondent at frequent intervals to monitor whether the delivery has occurred based on the expected date of delivery and conduct the first follow-up interview at approximately six weeks postpartum. In the event that the respondent does not have a phone or access to a phone, REs relies on the local Health Development Army (HDA), a group of community-based volunteers who monitor pregnant women in the community and assist Health Extension Workers (HEWs). In the case of pre-term/early deliveries, families are encouraged to inform the REs and HDAs. Once the 6-week interview is complete, the 6-month and 1-year interviews are scheduled. A week before each scheduled interview, the data management team sends an automated short text message in Amharic to each RE to remind them of the scheduled follow-up date. Data are collected within two to three weeks of the targeted interview date for each of the follow-up interviews. Women are initially approached for consent at the baseline interview, when REs describe the purpose of the study and read the approved consent language. At each follow-up interviews, women are asked if they have any questions and if they still agree to participate in the study, but the full consent language is not read again.

***SDP.*** All facilities identified as serving the EA through the sampling process described above are eligible for participation. A letter of support from regional and zonal health authorities is provided to all interviewers and consent is granted by the head of the facility or designated authority. In smaller facilities (e.g. health post, pharmacies, and drug shops), interviewees are generally the in-charge or owner of the facility. In larger facilities (e.g. hospitals, health centers), multiple respondents, generally the in-charge of each unit, provide information.

## Data management and availability

### Data management

Data are downloaded from the ODK Aggregate server daily and cleaned by the data management team using Stata version 16 ^12^. QGIS version 3.14 is used to review the distance between each household and the center of the EA to identify interviews that may have occurred outside of the study area ^22^.

When an error is suspected, the team communicates with the interviewer to confirm whether she entered the correct response, and in some cases to ask her to provide a correction. The team manually corrects these errors using Stata version 16 during data collection so that few errors remain by the end of field work.

### Weighting

Sample weights are constructed based on the selection probabilities of the EAs provided by the CSA. After data collection for each survey is complete, two weights (household and female) are created in the cross-section and two weights in the panel (household and female) to adjust for selection probability and non-response.

***Cross-section.*** The household weight is the inverse of the probability of selecting the EA multiplied by the probability of selecting the household within the EA. The weights are further adjusted to account for the non-response rate at the household level within the EA. As all females ages 15–49 years within selected households are surveyed, there is no further selection probability for the female. Instead to arrive at the final female weight, the household weight is adjusted for non-response of the female questionnaire among all eligible females within the EA. Female weights are created for de facto females only (i.e. women who slept in the household the night before the survey was administered). Application of the PMA-Ethiopia household and female survey weights for the cross-section result in a sample that is nationally representative of households and females age 15–49, respectively.

***Panel.*** As all households are included in the census, there is no additional selection probability of households; thus, the household weight is the inverse of the EA selection probability and the response rate to the census within the EA. Female weights for women in the panel are adjusted for non-response within the EA, and follow-up surveys will adjust for loss to follow-up from the baseline panel survey sample. Application of the PMA-Ethiopia household and female survey weights for the panel survey will result in a sample that is representative of all households with a pregnant or recently postpartum women and all pregnant or recently postpartum women age 15–49 residing in the six regions included in the PMA-Ethiopia panel, respectively.

***Service Delivery Point***. No weights are applied in the SDP survey as the sample is not selected probabilistically from a known sampling frame.

### Data availability

Anonymized microdatasets from PMA-Ethiopia will be made publicly available within approximately six months of the close of each phase of data collection (e.g. data from 6-week interviews will be made available six months after the final 6-week interview is completed). Datasets will be available for download via the www.pmadata.org website and will be available in csv, xls, and dta format. Data cleaning and release follow the same principles of the PMA2020 survey ^7^.

To contribute to ongoing efforts to improve coverage estimates ^6^, one GPS point per cluster will be made available, which will be randomly displaced following the same procedures used in other PMA surveys ^23^. All SDP surveys include a variable indicating which PMA-Ethiopia EAs fall within its catchment area, allowing for linkages between health facilities and households.

## Analysis and dissemination plan

### Statistical analysis

Preliminary data analysis will focus on computing frequencies, proportions, means or medians. Characteristics of survey participants will be described by computing frequencies and percentages for categorical variables. The initial analytic approach will be largely exploratory for data quality checking, including non-response/missingness and the extent of “don’t know” responses.

For both the cross-sectional and the longitudinal anlayses, descriptive analyses will examine proportions, tabulations, and summary measures (mean, median, range, etc.) for summarizing binary, categorical, and continuous variables, respectively. Subgroup analyses will be performed when it is feasible (e.g., adequate sample sizes in the contingency table cells). The descriptive analysis is particularly relevant for the coverage estimates of specific health services which are of primary importance for the FMoH (i.e. delivery in a health facility, receipt of postnatal care, receipt of specific vaccines, etc.).

Time to event analyses (life table and hazard models) will be used to assess time to uptake of postpartum family planning services, median time of exclusive breastfeeding and contraceptive discontinuation and switching within one year postpartum. Use of health services and receipt of specific components of care (i.e. receipt of postpartum family planning counseling, receipt of exclusive breastfeeding counseling) will be the primary independent variables to assess differences in utilization/duration, adjusted for confounding individual and partner characteristics.

We will conduct trend analysis to assess changes in vaccination completion rate per the vaccination schedule, modern contraceptive prevalence rate, contraceptive unmet need, and other high-impact intervention indicators (i.e. received treatment for maternal complications, immediate newborn care, etc.) over the four years of the study.

To assess the reliability and validity of the reproductive empowerment scale items, analysis of item performance, factor analysis, and association of the empowerment measure with specific outcomes will be evaluated. More detail is provided below.

*Analysis of item performance and factor analysis*: For the questions on reproductive empowerment, item-scale correlations, means, and variances will be computed. Internal consistency will be determined using Cronbach’s alpha coefficient ^24^. Principal Component Analysis (PCA), Parallel Analysis (PA), and Exploratory Factor Analysis (EFA) will be conducted based on polychoric correlation matrices. Factor analysis will also guide the selection of a more parsimonious set of items loading on the identified factors (based on 0.40 factor loading criteria) ^25^. Cronbach’s alpha values of 0.70 or higher will help determine the internal consistency of the subscale and scale items.

All analyses will be performed in Stata or R, per analyst preference. All statistical tests will be done at a significant level of p≤0.05. The data will be presented in text narratives, tables and graphical presentations.

## COVID-19

The COVID-19 pandemic has led to severe disruptions to everyday life in Ethiopia. A State of Emergency was instituted on April 8, 2020 that limited movement and transportation availability; while health seeking was not prohibited, it is not clear if and how coverage of key RMNH interventions was affected. Modeled estimates show that even minor disruptions in the coverage of key interventions could have critical impacts on maternal and newborn mortality ^26, 27^ but data on the true impact to service delivery are scarce.

The COVID-19 pandemic occurred approximately halfway through the data collection schedule for the 6-weeks interview; that is, approximately half of women enrolled in the panel gave birth before the State of Emergency was introduced and the remaining half will give birth after the State of Emergency was introduced. Comparisons between these groups will generate important information on whether rates of skilled birth attendance, postnatal care, and early childhood vaccinations have been impacted by disruptions resulting from the COVID-19 pandemic, including how these may have changed by sub-groups. Longer term outcomes, such as nutrition, vaccinations, and PPFP uptake can also be assessed and compared within the cohort and to external data sources to determine the extent of service delivery disruption. A short battery of questions assessing if and how the COVID-19 pandemic affected care-seeking for a range of behaviors was included in 6-week, 6-month, and 1-year surveys administered after April 2020.

### Dissemination of information

Key results from the cross-section, baseline, and SDP surveys will be presented in briefs and in PowerPoint presentations and made available at https://www.pmadata.org/countries/ethiopia. Results will include an overview of service coverage and comprehensiveness, family planning indicators, and availability of essential RMNH and FP commodities. Results related to delivery, postnatal care, nutrition, and vaccine coverage will be made available as follow-up interviews are conducted.

Findings from inferential analyses will be published in open-access, peer-reviewed journals. Findings of significant importance to the FMoH and PAB will be discussed at PAB meetings to inform policy and program response and identify future research trajectories.

### Study status

Data collection is ongoing, with the study scheduled to end in 2023.

## Limitations

Though PMA-Ethiopia is already an important data source, there are some limitations to its use that should be noted. In terms of the cross-section, PMA Ethiopia shares similar limitations to other cross-sectional surveys. Temporality cannot be assessed and thus causality cannot be established from associations. The indicator used to determine sample size was modern contraceptive prevalence rate and thus the sample size may not be adequate to measure other indicators with high precision.

As women self-report pregnancy status, it is likely that the panel underestimates both early pregnancies and pregnancies that end in abortion. Similarly, panel respondents are not representative of all women, but are representative of 90% of currently pregnant/recently pregnant women in Ethiopia. Data collected among all women, such as contraceptive use, adolescent health, and early marriage, are best explored using the cross-sectional data or with other data collection platforms.

Finally, the SDP survey cannot be treated as representative of all facilities within Ethiopia. Rather, these facilities, particularly when linked to the household information, are representative of the services available to a nationally representative selection of households. Future efforts will weight the sample to reflect the national distribution of health facilities.

## Conclusion

PMA-Ethiopia is an important new source of data for researchers, policy makers, and program implementors who wish to improve the RMNH continuum of care in Ethiopia. With the inclusion of cross-sectional, panel, and facility surveys, the project will provide estimates of coverage of key RMNH interventions, identify factors that are associated with care-seeking behaviors, and generate new data using innovative measurements in regular nationally-representative samples. Additionally, household and service delivery point data are gathered simultaneously and can be geographically linked which will improve effective coverage measurement and address many of the limitations that hinder current research efforts. Finally, the measurement platform will provide a critical source of data to measure changes in RMNH indicators as a result of the COVID-19 pandemic, including comparisons of service delivery coverage over one-year intervals and assess change in RMNH over time.

## Data availability

### Underlying data

No data are associated with this article.

### Extended data

All questionnaires are publicly available on the www.pmadata.org website.

The 2019 Service Delivery Point Questionnaire is available at https://doi.org/10.34976/kvvr-t814 ^13^

The Panel Questionnaire is available at https://doi.org/10.34976/h75w-8084 ^14^

The 2019 Cross-Sectional Household and combined Female Questionnaire is available at https://doi.org/10.34976/6hen-vd80 ^15^

To gain access to datasets when they become available you will need to create a profile and apply for access via this link - https://www.pmadata.org/data/available-datasets/request-access-datasets. When data collection is finished, all datasets will be made indexed and made available through the www.pmadata.org website.
