# Supplementary material for: Protocol for PMA-Ethiopia: A new data source for cross-sectional and longitudinal data of reproductive, maternal, and newborn health
Source: Gates Open Res. 2020 Sep 9;4:126. [Version 1] doi: 10.12688/gatesopenres.13161.1 (PMC7593701; doi:10.12688/gatesopenres.13161.1)
Supplement: Peer review history

# Peer review: Reviewer response for version 1

**DOI:** 10.21956/gatesopenres.14358.r29832

**Published:** 2020-10-28

**Authors:** Jennifer Winston

**Affiliations:** 1 Carolina Population Center, University of North Carolina at Chapel Hill, Chapel Hill, NC, USA

This is a very helpful document detailing the protocol used in collecting the three PMA-Ethiopia surveys. As the PMA surveys conducted in Ethiopia differ from PMA surveys conducted in in other countries and in other rounds, this documentation is helpful for researchers using these data and for others interested in survey design. Their ongoing data collection during the COVID-19 pandemic also provides a unique opportunity to assess the impact of the pandemic on RMNH.

 My one suggestion would be to clarify which SDPs are included in the SDP survey. Selection of private facilities is well described, but it is not clear if all public facilities are included.

Is the study design appropriate for the research question?

Yes

Is the rationale for, and objectives of, the study clearly described?

Yes

Are sufficient details of the methods provided to allow replication by others?

Yes

Are the datasets clearly presented in a useable and accessible format?

Not applicable

Reviewer Expertise:

Medical geography, family planning

I confirm that I have read this submission and believe that I have an appropriate level of expertise to confirm that it is of an acceptable scientific standard.

# Peer review: Reviewer response for version 1

**DOI:** 10.21956/gatesopenres.14358.r29760

**Published:** 2020-10-21

**Authors:** John Cleland

**Affiliations:** 1 Faculty of Epidemiology and Population Health, London School of Hygiene and Tropical Medicine, London, UK

PMA-Ethiopia has already shown itself to be a critically important data source for monitoring MCH/FP progress. This protocol presents a detailed description of the three components: the nationally representative cross-sectional survey; the panel study in six regions focused on maternity and child care and health from ANC through the 12 postpartum months; and the survey of service delivery points. The availability of data collection instruments was welcome and the use of scales such as the London Measure of Unintended Pregnancy represents the potential for improved understanding beyond what can be derived from Demographic and Health Surveys.

 I have three queries and two suggestions:

1. The sample design describes selection of EAs with probability proportionate to size followed by a fixed selection of 35 households per EA. This should yield a self-weighting sample provided that the measures of size are accurate. Yet the protocol mentions weighting the data to take account of differing selection probabilities. Is this needed to adjust for inaccuracies in measures of EA sizes?
2. Ethiopia is a multi-lingual country. Do you prepare different linguistic versions of the data collection instruments or do you expect interviewers to attempt spontaneous translations for those respondents who are not fluent in Amharic?
3. Loss to follow-up in the panel survey is a critical issue and it is unclear whether, or how, you plan to follow-up women who migrate out the selected cluster.

My two suggestions:

1. It is well established that many women await the return of menses before adopting contraception. I was unconvinced that the information in the panel study collected sufficiently detailed information to illuminate this topic.
2. I wonder whether you need to repeatedly collect attitude data as in section 6, as they are unlikely to change over short time periods.

Is the study design appropriate for the research question?

Yes

Is the rationale for, and objectives of, the study clearly described?

Yes

Are sufficient details of the methods provided to allow replication by others?

Yes

Are the datasets clearly presented in a useable and accessible format?

Not applicable

Reviewer Expertise:

fertility, family planning,child survival

I confirm that I have read this submission and believe that I have an appropriate level of expertise to confirm that it is of an acceptable scientific standard.
